# Management of Heart Failure, Durable Left Ventricular Assist Device, and Heart Transplant Patients in the COVID-19 Era

**DOI:** 10.14797/mdcvj.651

**Published:** 2021-12-15

**Authors:** Nadia Fida

**Affiliations:** 1 Houston Methodist Hospital, Houston, Texas, US

**Keywords:** COVID-19, heart failure, heart transplant, left ventricular assist device

## Abstract

Our world is facing recurrent waves of coronavirus disease 2019 (COVID-19) with the emergence of more infectious strains of the novel coronavirus, SARS-CoV-2. Multiple studies have established that heart failure (HF) patients are at high risk of severe disease and poor outcomes with COVID-19. Management of COVID-19 in patients with HF, heart transplant, and those supported with durable left ventricular assist devices present an arduous challenge due to underlying complex health conditions and overlap of symptoms. Based on available data, this review outlines the management of this vulnerable patient population who either present with COVID-19 with preexisting HF or with de novo HF from COVID-19.

## Epidemiology of COVID-19 in Heart Disease

Since December 2019, when SARS-CoV-2 was first identified in Wuhan, China, over 250 million cases have been reported worldwide. As of this writing, we are in the midst of the fourth surge with the highly infectious and transmissible Delta variant and the new Omicron variant starting to emerge. This pandemic has tested the capacity of healthcare delivery worldwide while also affecting the global economy.

The elderly and people with underlying chronic conditions such as asthma, cardiovascular disease, and immune system disorders are at particular risk for poor health outcomes. In a state-of-the-art review published in the *Journal of the American College of Cardiology*, Driggin et al. highlighted a number of studies, including two large population-based studies, suggesting an association between cardiovascular (CV) risk factors and underlying CV conditions in the COVID-19 cohorts. The authors found that increased case fatality rates and intensive care admissions were more likely in patients who have a higher comorbidity burden for cardiovascular risk factors than those who do not. For instance, although the overall case-fatality rate among 44,672 confirmed COVD-19 patients in Wuhan was 2.3%, it was higher in those with cardiovascular disease (10.5%), diabetes (7.3%), and hypertension (6.0%).^1^ Several studies have shown that myocardial injury in COVID-19 can be direct or indirect, but regardless of the underlying mechanism, troponin elevation in COVID-19 carries prognostic value in predicting both clinical severity and mortality.^2^ Additionally, the risk appears to be continuous, with higher elevations predicting worse outcomes.^3^

## COVID-19 Mechanisms of Cardiac Injury

**Figure 1:**
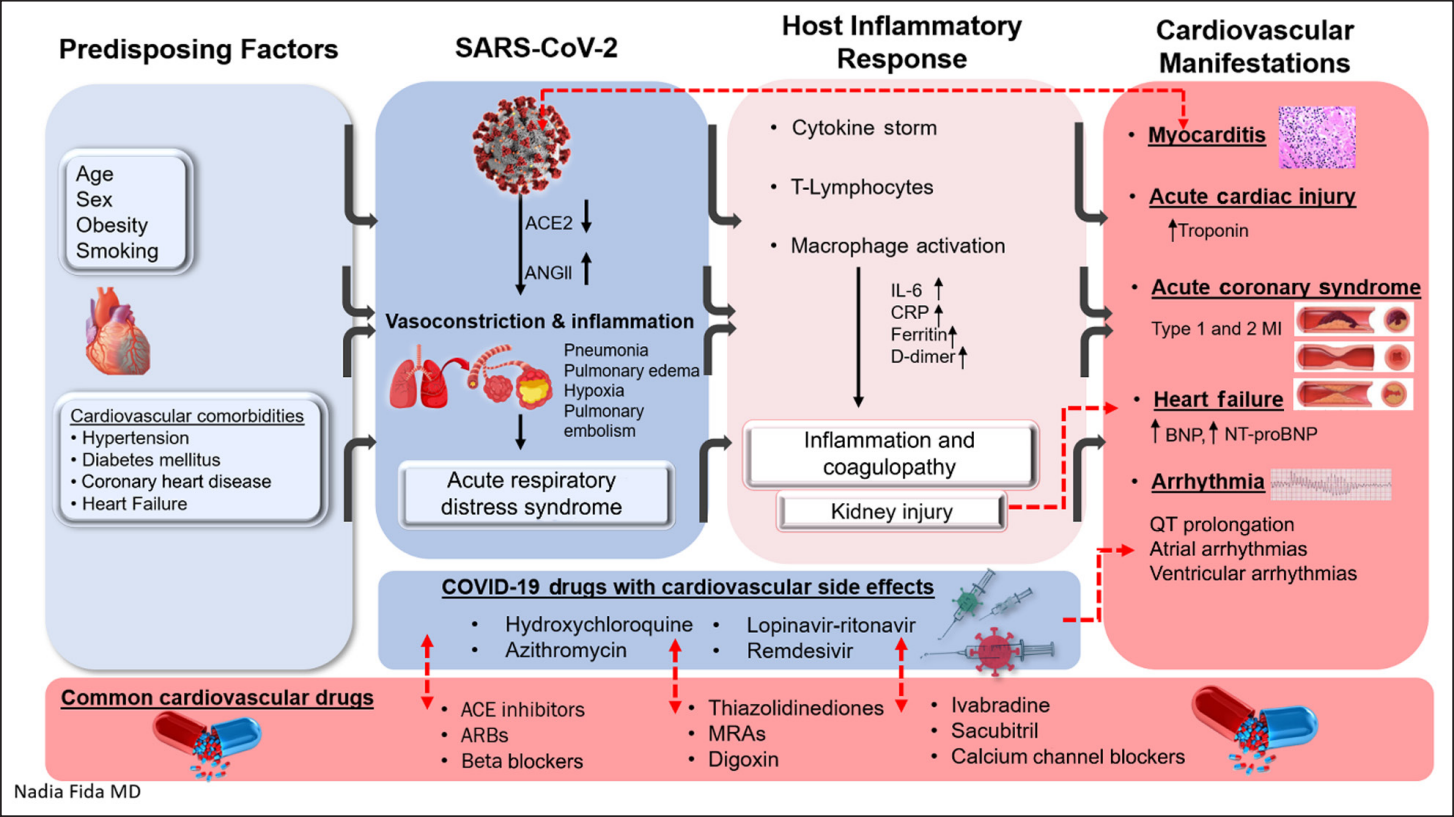
Interfaces of mechanisms of injury in heart failure and COVID-19. ACE2: angiotensin-converting enzyme 2; ANGII: angiotensin 2; ACE: angiotensin-converting enzyme; ARBs: angiotensin receptor blockers; CRP: C-reactive protein; MI: myocardial infarction; BNP: B-type natriuretic peptide; NT-proBNP: N-terminal pro-BNP

To understand the impact of COVID-19 in heart failure (HF), it is important to understand how the virus acts and its mechanisms of injury (***Figure 1***). SARS-CoV-2 infection is triggered by the binding of the S protein to its functional receptor angiotensin-converting enzyme 2 (ACE2) on the surface of host cells, typically via a type II pneumocyte. This viral complex is incorporated into the cytoplasm either by direct fusion with the cell membrane or via endocytosis. This leads to ACE2 down regulation, which activates the de novo renin angiotensin aldosterone system (RAAS) and causes an intense inflammatory response, increased pulmonary vascular permeability, and pulmonary edema.

This mechanism prompted earlier preclinical studies to suggest that RAAS inhibitors (commonly used drugs in HF) might increase susceptibility to infection and patients should discontinue their use. However, it has been reported that the serum level of angiotensin II is significantly elevated in COVID-19 patients and correlates directly with viral load and lung injury.^4^ Thus, continuation of ACE inhibitors (ACEIs) and angiotensin receptor blockers (ARBs) might prevent widespread endothelial dysfunction and varying degrees of multiorgan damage resulting from the RAAS activation. In patients with HF, the process of reverse remodeling via neurohormonal modulation exploits the wide expressivity of ACE2 on cardiac myocytes, fibroblasts, and coronary endothelium to block RAAS, promote cardioprotective vasodilating properties, and decrease myocardial hypertrophy and fibrosis. This interaction between the S protein and ACE2 has also offered a therapeutic target for this syndrome.

Multiple pathophysiological pathways are implicated in the diverse cardiovascular manifestations of COVID-19. Infection with SARS-CoV-2 results in intense cytokine storm and a proinflammatory state with hypercoagulability, increased sympathetic stimulation with vasoconstriction, hypoxic injury, sepsis, and, in severe cases, acute respiratory distress syndrome (ARDS) and multiorgan failure. These various insults lead to myocardial injury, HF, acute coronary syndromes (ACS), arrythmias, and thromboembolism. However, evidence of direct myocardial injury remains sparse.

Cardiac myocytes are a potential target for SARS-CoV-2 due to the virus’ cell entry mechanism.^5^ Myocarditis has been reported in a limited series from China, where 7% of deaths were attributed to myocardial damage but without a clear, definite diagnosis of myocarditis. Similarly, other reports have described fulminant myocarditis in the setting of high viral load with autopsy findings of inflammatory mononuclear infiltrate in myocardial tissue, but without histologic evidence of direct COVID-19 infection in the myocardial cells.^6,7,8^ Thus, increases in cardiac troponin indicative of myocardial injury are common and prognostic in COVID-19,^9^ albeit they represent multiple etiological mechanisms of cardiac injury that must be correlated with a clinical picture and other diagnostic modalities. Evidence to date suggests that direct myocardial injury is plausible but largely attributable to indirect effects of severe systemic inflammation. Direct cellular injury of myocyte is reported in autopsy findings by Bailey et al., who examined four autopsies and demonstrated cardiac myocyte infection by identifying intracellular viral particles budding on electron microscopy with associated macrophage infiltration and cell death. ***Figure 1*** outlines interfaces of various mechanisms of injury in HF and COVID-19.^10^

## Heart Failure and COVID-19

Studies to date have reported a variable prevalence of HF in patients with COVID-19. Moreover, it is difficult to elucidate the prevalence of a decompensated state of chronic HF from COVID-19 versus incidence of acute de novo HF from the viral syndrome. Shi et al.^9^ reported an HF prevalence of 4% in a cohort of patients hospitalized with COVID-19 at a single center in Wuhan, with similar percentages for cardiomyopathy among hospitalized COVID-19 patients in a study by Guo et al.,^11^ whereas other studies from Wuhan reported HF incidence in up to a quarter of studied patients and up to 50% of those who died.^12,13^ Older age and a high burden of comorbid conditions, especially in patients with HF with preserved ejection fraction, predispose patients to a decompensated state in the setting of sepsis, heightened inflammatory response, and impaired renal axis.

### Acute Heart Failure

Acute HF may complicate the clinical course of severely ill COVID-19 patients. Prolonged and worsening hypoxemia can lead to hypoperfusion, multiorgan failure, and worsening hemodynamics. The interplay of several mechanisms in COVID-19 with acute HF can exacerbate clinical worsening, possibly leading to acute myocardial ischemia from type 1 or type 2 myocardial infarction or myocarditis, ARDS, acute kidney injury, stress-induced cardiomyopathy, and tachyarrhythmias. A profound systemic cytokine storm characteristic of COVID-19 also contributes to acute HF or exacerbates chronic HF in COVID-19. Patients who present with ACS due to type 1 or type 2 myocardial infarction in the setting of COVID-19 are susceptible to a decline in contractile function and decompensation of underlying disease. Furthermore, a prolonged hospital stay for severely ill COVID-19 patients with advanced sepsis, cytokine storm, and immune response to infection makes them susceptible to developing stress-induced cardiomyopathy.^14^ Regardless of underlying pathophysiological response, decompensated HF worsens underlying pulmonary edema associated with ARDS and, in severe cases, leads to other multiorgan impairment due to elevated filling pressures with or without hypoperfusion.

### Chronic Heart Failure

Patients with underlying HF are at increased risk of severe COVID-19 illness, and a high index of suspicion for viral testing should be maintained for this vulnerable population. Telemedicine should be considered whenever possible to provide medical advice and follow-up of stable HF patients to avoid exposure, and vaccination should be encouraged and reinforced.

## Management of Patients with Heart Failure and COVID-19

### Preventive Measures

Since patients with HF are at much greater risk of morbidity and mortality with COVID-19, it is imperative that appropriate measures are taken by healthcare professionals to ensure safety while providing high-quality care for these patients. ***Table 1*** outlines effective patient management steps to enhance safety and mitigate risk.

**Table 1:** Recommendations for heart failure (HF) patient management to enhance safety and mitigate risk in the setting of COVID-19. MCS: mechanical circulatory support; IABP: intra-aortic balloon pump; ECMO: extracorporeal membrane oxygenation; VAD: ventricular assist device; LVAD: left VAD; INR: international normalized ratio.

RISKS	RECOMMENDATIONS

Patients with HF are at high risk of major complications HF may predispose to pulmonary complications in COVID-19 COVID-19 directly and indirectly increases the risk of cardiovascular complications	All HF patients should receive vaccination Guideline-directed medical therapy for HF should be continued when possible Beware of drug-to-drug interactions Multidisciplinary decision making is required for patients in shock needing MCS – IABP, temporary VAD or ECMO LVAD patients with COVID-19 need close monitoring of INR because of COVID-19–associated coagulopathy Heart transplant patients with COVID-19 need their immunosuppression regimen adjusted, monitoring for hematological abnormalities, and close follow-up

Low-risk ambulatory patients, especially immunocompromised patients, should be encouraged to get vaccinated and observe appropriate masking in healthcare settings to prevent exposure. All healthcare personnel should maintain proper personal protective equipment and undergo surveillance testing according to institutional protocols. Virtual encounters are encouraged to help determine if an in-person clinic visit is required and also to reassure and educate patients to reduce their anxiety and ensure that they seek health care when necessary. In-person visits should be completed for high-risk patients, and those who fail COVID-19 screening should be seen virtually.

### Evaluation of Patients with Heart Failure and COVID-19

It is extremely important to obtain a thorough history and physical examination because COVID-19 and HF can present with overlapping symptoms and signs related to the cardiopulmonary involvement. In the face of recurrent surges and emergence of highly infectious strains, physicians must keep a high index of suspicion that COVID-19 is the cause of HF decompensation. Similarly, patients who have underlying cardiovascular disease and its risk factors are at greater risk for worsening COVID-19 syndrome and poor outcomes. History suggestive of exposure to COVID-19, fever, upper and/or lower respiratory tract infection symptoms, loss of taste and smell, and fatigue are all signs of COVID-19 infection, although fever is observed in less than half of patients.^15^ Elevated jugular venous pressure, rales, and edema are more suggestive of HF, which in itself can exacerbate underlying pulmonary edema. Most patients with severe COVID-19 develop hypoxia 5 to 10 days after onset of infection, which can then rapidly progress to ARDS and aggravate preexisting organ dysfunction leading to multiorgan failure.^16,17^

Electrocardiogram abnormalities are highly prevalent in hospitalized COVID-19 patients, mostly as nonspecific ST-T changes. In a study of 138 patients with COVID-19 admitted to a hospital in Wuhan, 16.7% had arrythmia.^18^ Similarly, in a multicenter international registry, 14% of hospitalized COVID-19 patients developed QT prolongation by 7 days after admission.^19^ Underlying cardiac injury, proarrhythmic potential of medications used in management of COVID-19, and electrolyte abnormalities all require close monitoring. Computed tomography provides high sensitivity and specificity to diagnose COVID-19–associated lung disease^20^ and pulmonary embolism, and it can provide information on the extent of complications like superimposed infections, effusions, and edema.

Multiple laboratory abnormalities are seen in patients with COVID-19. Numerous reports indicate that severity of COVID-19 is inversely correlated with low lymphocyte count, with repletion linked to recovery. Extracorporeal membrane oxygenation (ECMO) can exacerbate lymphocyte depletion.^21^ Brain-type natriuretic peptide (BNP) is elevated in many patients with COVID-19 and HF; however, a low level of BNP has a very high negative predictive value for excluding cardiac dysfunction. As discussed, troponin elevation (illustrating myocardial injury) is common among hospitalized patients with COVID-19 (incidence ranging from 14–44% across 18 studies), with higher levels correlated with worse outcomes.^2^ Increases in markers of inflammation and thrombogenicity such as C-reactive protein, erythrocyte sedimentation rate, ferritin, interleukin-6, lactate dehydrogenase, fibrinogen, and D-dimer are associated with higher mortality.^18,22^

**Figure 2:**
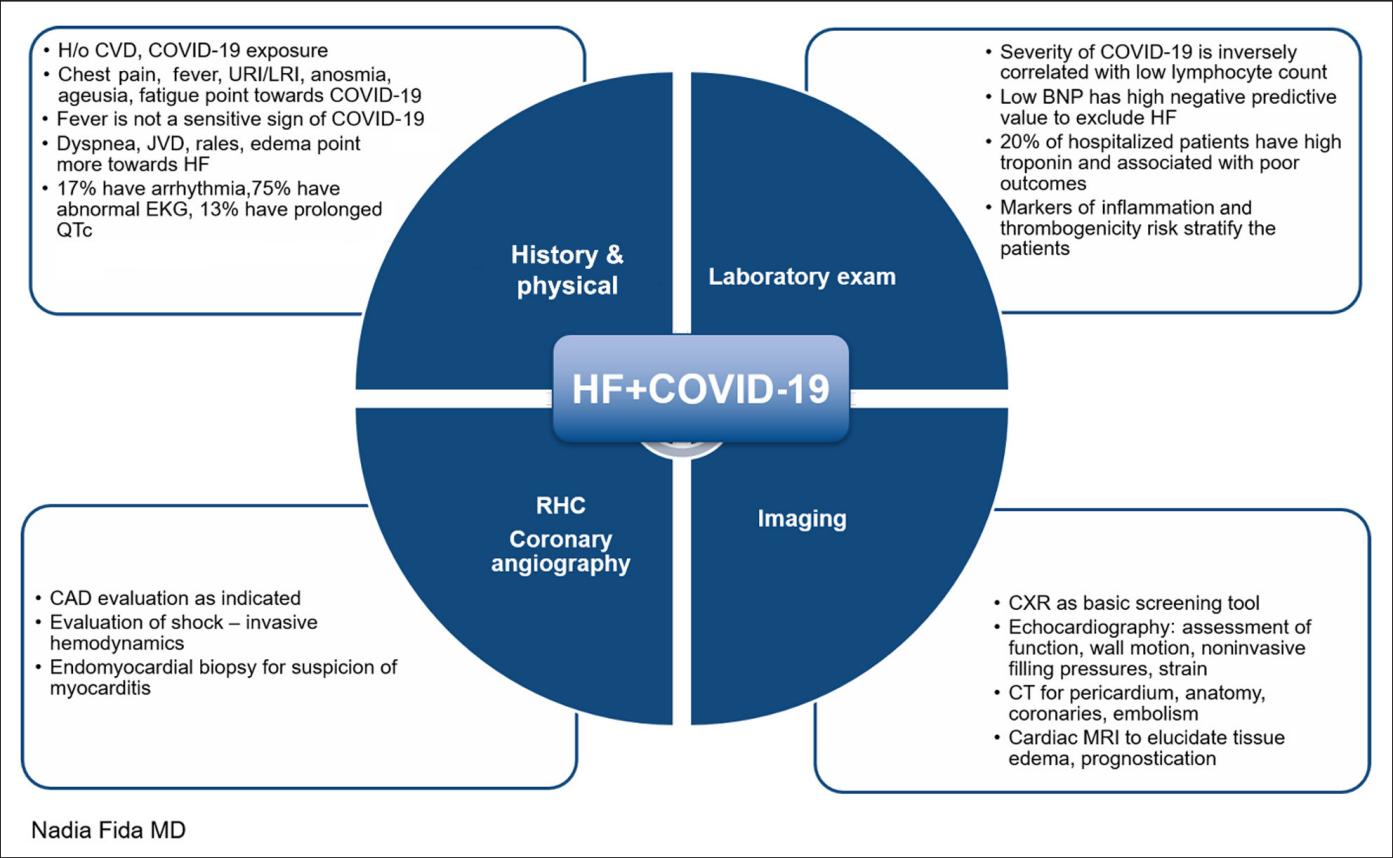
Evaluation of patients with heart failure (HF) and COVID-19. CVD: cardiovascular disease; URI/LRI: upper respiratory tract infection/lower respiratory tract infection; JVD: jugular vein distention; EKG: electrocardiogram; CAD: coronary artery disease; BNP: B-type natriuretic peptide; CXR: chest x-ray; CT: computed tomography; MRI: magnetic resonance imaging; RHC: right heart catheterization

Transthoracic echocardiography (TTE) is an important screening and diagnostic tool and must be considered in all patients with HF, heart transplant (HT), or left ventricular assist devices (LVADs) with suspected or confirmed COVID-19. TTE provides assessment of biventricular cardiac function and filling pressures, allows noninvasive hemodynamics evaluation, and helps distinguish concomitant causes of HF, either preexisting (eg, valve disease) or COVID-19–related (eg, right ventricular dysfunction secondary to pulmonary embolism) complications. Patients who present with rising troponin may need coronary angiography. Invasive hemodynamics may need to be assessed in patients presenting with shock. When available, cardiac magnetic resonance imaging should be used when myocarditis is suspected.^23^ Endomyocardial biopsy should be considered for patients presenting with de novo HF and/or myocarditis.***Figure 2*** outlines assessment of patients with HF and COVID-19.

### Treatment of Patients with Heart Failure and COVID-19

Recurrent surges in the COVID-19 pandemic have led to hospital bed and staff shortages, necessitating the use of telemedicine to monitor stable patients at home. HF patients, including those with HT or LVAD, remain at high risk of complications and mortality when infected by SARS-Cov-2.^12,24^ Appropriate consultants (eg, pulmonologists, nephrologists, infectious disease specialists, and pharmacists) should be involved early to manage other organ systems, immunosuppression, and drug interactions. Palliative consultation should be obtained for very sick patients with low likelihood of recovery.

**Medications.** Hospitalized HF patients should be continued on guideline-directed medical therapy (GDMT) as much as possible depending on their clinical stability, underlying renal function, and pulmonary status. Some maintenance of diuretics is helpful for pulmonary decongestion, even when patients appear to be euvolumic. Patients should be closely monitored for signs of hypoperfusion, and medications should be withdrawn as needed. ACEIs, ARBs, angiotensin receptor-neprilysin inhibitors, and mineralocorticoid antagonists (MRAs) should be continued unless clinically contraindicated due to hypotension, acute kidney injury, or hyperkalemia. Several large series have confirmed that ongoing treatment with ACEIs/ARBs does not increase the severity of the clinical course of infection^25^ and may actually confer protection from severe COVID-19–related illness.^26^ A randomized clinical trial (RCT) in Brazil enrolled 659 HF patients hospitalized with mild-to-moderate COVID-19 who were taking ACEIs or ARBs before hospital admission and found no significant difference in the mean number of days alive and out of the hospital for those who discontinued versus continued these medications.^27^ These findings do not support routinely discontinuing ACEIs or ARBs unless clinically contraindicated. Beta blockers should also be continued unless the patient is in a low-output state. They can be helpful in sinus and atrial tachyarrhythmias. Treatment with intravenous metoprolol tartrate was observed to improve respiratory status in a small randomized trial of patients with COVID-19 and ARDS requiring mechanical ventilation.^28^ Some antiviral medications used in the treatment of COVID-19 may cause bradycardia, necessitating dose reduction or withdrawal. Ivabradine can be helpful to control heart rate when beta blockers cannot be used because it does not affect inotropy.^20^

**Extracorporeal membrane oxygenation.** Patients with worsening hypoxemia and/or circulatory shock unresponsive to standard management should be assessed for candidacy for veno-venous (V-V) or veno-arterial (V-A) ECMO. COVID-19 patients whose clinical course is complicated by refractory ARDS necessitating ECMO usually stabilize with V-V cannulation unless they have a high vasopressor requirement. Less commonly, patients with myocarditis, stress-induced cardiomyopathy, or ACS may require V-A ECMO. ECMO duration may be longer in patients with COVID-19 than in those without, but published data shows similar mortality between the two groups.^29^

**Figure 3:**
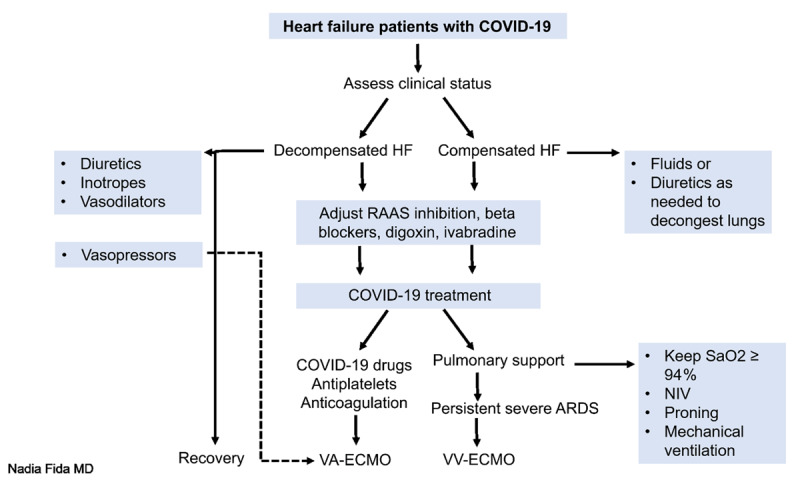
Suggested algorithm for the management of patients with heart failure (HF) and COVID-19. VA-ECMO: veno-arterial extracorporeal membrane oxygenation; VV-ECMO: veno-venous; NIV: noninvasive ventilation; RAAS: renin-angiotensin-aldosterone system; ARDS: acute respiratory distress syndrome; SaO2: arterial oxygen saturation

**Anticoagulation.** Both HF and COVID-19 syndrome are hypercoagulable states. Long-term anticoagulation should be continued in less sick patients but switched to low-molecular-weight heparin in sicker patients due to drug interactions with novel anticoagulants. All hospitalized patients with COVID-19 should receive pharmacologic thromboprophylaxis unless the risk of bleeding outweighs the risk of thrombosis. In the setting of heparin-induced thrombocytopenia, fondaparinux is recommended. Therapeutic and intermediate-dose anticoagulation are being studied to reduce mortality and/or severity of COVID-19 in multiple RCTs. However, given the variability in interventions and primary outcome measures, it remains uncertain whether higher-intensity anticoagulation offers benefit over standard thromboprophylaxis in patients hospitalized for COVID-19, particularly in moderately ill patients.^30,31^ Recently published data from a large RCT sponsored by the National Institutes of Health showed that, in critically ill patients with COVID-19, an initial strategy of therapeutic-dose anticoagulation with heparin was no more effective than usual-care pharmacologic thromboprophylaxis in improving survival to hospital discharge or the number of days free of cardiovascular or respiratory organ support.^32^ ***Figure 3*** presents a suggested algorithm for managing patients with HF and COVID-19.

## Considerations for Patients on Long-Term Mechanical Circulatory Support

Patients on long-term mechanical circulatory support (MCS) have an inherently higher risk of infections, mainly due to hardware, drive line exit site, and functionally impaired immunity. Moreover, warfarin, which is notorious for interactions, is the only approved anticoagulant for this patient population. Therefore, extensive preventive measures should be employed against COVID-19 for this cohort of patients. The Trans-CoV-VAD registry, a multicenter registry of LVAD and cardiac transplant patients with confirmed COVID-19 in the United States, includes data on 40 patients and is the largest LVAD series reported to date. They report no significant differences in the racial or gender breakdown of COVID-19 cases compared with the total population receiving LVADs in the last 21 months at participating sites (N = 955). Of the 40 COVID-19–positive patients, 60% were hospitalized, 25% developed critical illness, and 1 patient had a suspected LVAD thrombosis that was medically managed. Overall, the case fatality rate was high at 20%, with death occurring at a median of 22 days after admission.^33^

Advanced HF patients who need an LVAD implant are particularly vulnerable in their perioperative period. These patients might face longer recovery times due to limited available rehabilitation options.

## Consideration for Patients with Heart Transplant

Patients with HT may be at increased risk of mortality from COVID-19 due to comorbidities and immunosuppression. Precautionary measures of social distancing, masking, sterility, and balancing the need for patient visits against risk of infection are fundamental to limit COVID-19 illness among HT recipients. In a multicenter prospective study in Spain, demographics, clinical features, management, and outcomes in solid organ transplant recipients diagnosed with COVID-19 were compared before (first wave) or after (second wave) July 13, 2020. Of these, 149 (9%) were HT recipients; they had a case-fatality rate of 19.7% in the first wave and 14.5% In the second.^34^ Multicenter data from northern Italy show a doubling of COVID-19 prevalence and related mortality (30%) for patients with HT. However, this increased mortality may be related to the speed at which the pandemic spread in northern Italy, straining the healthcare system.^35^ A study from a large transplant center in New York City captured a similar case-fatality rate.^36^

A recently published study including 99 HT patients with COVID-19 from 11 centers reported observations regarding the clinical course, immunosuppression, and outcomes. The median time from transplant to infection was 5.6 years. Age ≥ 60 years was associated with a higher risk of death, and use of a combination of calcineurin inhibitor, antimetabolites, and prednisone was associated with more severe disease compared with the combination of calcineurin inhibitor and antimetabolite alone (adjusted OR = 7.3; 95% CI, 1.8-36.2). Tachypnea, oxygen requirement, elevated creatinine, and inflammatory markers were predictive of a severe course. Interestingly, 43% of patients had no fever, but gastrointestinal symptoms were common, occurring in 46% of patients. Moreover, the investigators found a high case-fatality rate of 24% among hospitalized patients and 16% among symptomatic patients.^37^

Guidance from the International Society for Heart & Lung Transplantation (ISHLT) released in February 2021 states management for HT patients with COVID-19 should generally be the same as that for the general population, with some special considerations. For instance, patients with VADs may be proned, but care should be taken to avoid tugging the driveline, and driveline dressings can be changed when the patient is not prone. The ISHLT also advises that providers consider holding mycophenolate mofetil, mTOR inhibitors, or azathioprine in HT patients admitted with moderate-to-severe COVID-19 illness.^38^ Though practices vary, most transplant centers routinely deintensify immunosuppression in the face of active infection.

### COVID-19 Vaccination for Heart Failure and Heart Transplant Patients

Both the American Society of Transplantation and the ISHLT recommend vaccination of patients with HF and HT recipients and support a third dose for those who received an mRNA vaccine.^39^ Although it is preferred that patients would complete the vaccine series 2 weeks or more prior to transplantation, transplantation should not be routinely delayed. Updated recommendations suggest that transplant recipients should wait at least 1 month after transplant surgery to receive the COVID-19 vaccine. However, COVID-19 vaccination should be postponed for at least 3 months following T-cell or B-cell–depleting therapies (such as antithymocyte globulin, rituximab, etc.), whether given for induction immunosuppression or as treatment for rejection.^40^

Observational data has established that the COVID-19 vaccine is effective at reducing symptomatic disease, hospitalization, and death from COVID-19.^41,42,43^ While the detectable serological response is only about 50% in fully vaccinated solid organ transplant recipients (SOTRs), early clinical effectiveness data suggests that < 1% of fully vaccinated SOTRs experience breakthrough infections.^44,45^

## Conclusions

The COVID-19 pandemic represents a daily challenge for healthcare systems. Patients with HF, HT, and LVAD represent a high-risk cohort for developing COVID-19 and experiencing worse outcomes. Strict preventive measures and vaccinations are crucial to reduce the spread. Clinicians must be vigilant and maintain a high degree of suspicion in ruling out COVID-19 in hospitalized patients with HF, HT, and LVAD. GDMT should be continued in chronic HF patients whenever hemodynamic conditions and other organ function permit and considering drug interaction with COVID-19–related therapies and side-effect profiles. It is important to emphasize that as the pandemic evolves, so too does our understanding of the disease; therefore, the aforementioned recommendations may change as updated information is published.

## Key Points

- The COVID-19 pandemic with recurrent surges has tested the capacity of healthcare delivery worldwide. Numerous databases have established that the elderly and people with underlying chronic conditions, including cardiovascular disease, are at particular risk for poor health outcomes.
- Patients with heart failure (HF), including those with heart transplant and left ventricular assist devices, are at much greater risk of morbidity and mortality when infected with COVID-19. It is imperative that healthcare providers take appropriate preventive measures to ensure safety while providing high-quality care for these patients.
- As in the general population, the decompensated state of chronic HF from COVID-19 is a more common presentation than acute de novo HF. However, it remains challenging to tease out the true incidence of either manifestation. Based on available data, this review outlines the management of this vulnerable patient population who present with either COVID-19 with preexisting HF or with de novo HF from COVID-19.
